# A Micro Aerosol Sensor for the Measurement of Airborne Ultrafine Particles

**DOI:** 10.3390/s16030399

**Published:** 2016-03-18

**Authors:** Chao Zhang, Rong Zhu, Wenming Yang

**Affiliations:** 1 State Key Laboratory of Precision Measurement Technology and Instrument, Department of Precision Instrument, Tsinghua University, Beijing 100084, China; zhangchao14@mails.tsinghua.edu.cn; 2 School of Mechanical Engineering, University of Science and Technology Beijing, Beijing 100083, China; wmyang@ustb.edu.cn

**Keywords:** ultrafine particles, number concentration, particle size, micro aerosol sensor

## Abstract

Particle number concentration and particle size are the two key parameters used to characterize exposure to airborne nanoparticles or ultrafine particles that have attracted the most attention. This paper proposes a simple micro aerosol sensor for detecting the number concentration and particle size of ultrafine particles with diameters from 50 to 253 nm based on electrical diffusion charging. The sensor is composed of a micro channel and a couple of planar electrodes printed on two circuit boards assembled in parallel, which thus integrate charging, precipitating and measurement elements into one chip, the overall size of which is 98 × 38 × 25 mm^3^. The experiment results demonstrate that the sensor is useful for measuring monodisperse aerosol particles with number concentrations from 300 to 2.5 × 10^4^ /cm^3^ and particle sizes from 50 to 253 nm. The aerosol sensor has a simple structure and small size, which is favorable for use in handheld devices.

## 1. Introduction

Airborne nanoparticles or ultrafine particles [1,2] distributed in the atmospheric, indoor and industrial environments seriously threaten human health [3,4]. The number concentration and particle size are the two key parameters used to describe exposure to airborne nanoparticles or ultrafine particles. The toxicology research results show that aerosol particles can deposit in different parts of the human respiratory organs [4,5,6,7] according to the sizes of the particles. The particles with sizes of less than 10 µm can enter the nasal cavity, those smaller than 7 µm can enter the throat, and if less than 2.5 µm, they enter the lungs. Nanoparticles or ultrafine particles can enter into the human lungs and alveolar area, and further enter into the human blood circulation system [8,9].

Measurements of the size and concentration of aerosol particles mainly involve two kinds of methods based on optical and electrical mechanisms [1]. Optical measurements require a sensor or a particle detector in the detection zone; three of the most widely used sensors are the optical particle counter (OPC) [10], the laser particle counter (LPC) [11], and the condensation particle counter (CPC) [12]. However particle size detection by light scattering loses sensitivity when the size is less than the wavelength of the light or laser used, so OPCs or LPCs can only detect particle sizes larger than 0.1 µm [1]. CPCs can detect particles with sizes less than 0.1 µm, but to date the limitations of their compactness, portability and cost do not allow their application for personal monitoring. The particles with sizes ranging from 1 nm to 300 nm can be detected by electrical measurement. Electrical measurements can be classified into two groups, according to their specific measurement principle. One, exemplified by the Scanning Electrical Mobility Spectrometer (SEMS) [13] or Differential Mobility Analyzer (DMA) [14] techniques, is based on the fact that the electric mobility of charged particles is inversely proportional to the particle size. However, these instruments cannot be handheld due to their big volume and heavy weight. The other measurement methods are based on diffusion charging whereby the average charge on particles corresponds roughly with their diameter in a certain size range [1]. The aerosol particles are charged by gas ions which are ionized in a specific charger, and then excess gas ions unattached to the aerosol particles are removed so as not to affect the subsequent current measurement, and finally the number concentration and the particle size can be calculated according to the charges measured on the particles. Some handheld instruments based on the charging principle have been reported, such as the Nanomonitor [15,16], NanoCheck (Model 1.320, GSI), and Discmini [17,18,19] devices. The Nanomonitor can measure number concentrations of ~10^6^ /cm^3^ and averaged particle sizes between 10–300 nm, the its configuration mainly consists of three sections, *i.e.*, charging, precipitation and sensing. It measures the charge current using a block-shaped voltage that varies between low and high voltages. At the low voltage, the excess ions are removed, while at the high voltage, a part of the charged particles are removed as well. A current meter which is connected via a Faraday cage records two different currents under low and high precipitating voltages. The particle parameters of number concentration and size can be figured out from the detected currents. The NanoCheck uses a variable ion trap voltage to obtain particle parameters which can measure particles in the size range of 25–300 nm and number concentrations of 500–5 × 10^5^ /cm^3^ based on the same operating principle as the Nanomonitor. The Discmini [19] can measure averaged particle sizes between 15–400 nm and number concentrations from 700 to 8.4 × 10^5^ /cm^3^. The aerosol particles are electrically charged in a corona discharger and detected in two stages. Small particles are deposited in the diffusion stage with a stack of stainless steel screens, while larger particles pass through the diffusion stage and are detected in the filter stage which contains a HEPA filter. The Nanomonitor, the NanoCheck and the Discmini all use Faraday cages, ion-traps and/or a stack of stainless steel screens to detect the charge currents which have elaborate structures, hindering further miniaturization.

Another kind of particle detector is based on a resonant cantilever [20,21,22]. Wasisto *et al.* designed an airborne nanoparticle detector based on a microelectromechanical (MEMS) silicon resonant cantilever which can only detect the aerosol mass concentration [20]. The cantilevers were fabricated by using silicon bulk MEMS processes. Some new chargers fabricated by using nanomaterials have emerged as well. Hwang *et al.* designed a ZnO nanowire charger [23] for aerosol particle charging.

In this study, we developed a micro aerosol sensor, which can detect monodisperse aerosol particle number concentrations in the range of 300–2.5 × 10^4^ /cm^3^ and particle sizes in the range of 50–253 nm based on the diffusion charging principle. The aerosol sensor consists of three essential sections, the charging, precipitation and measurement sections. The three sections are integrated into one chip, composed of a couple of planar electrodes printed on two parallel-assembled circuit boards rather than elaborate structures, such as ion traps and Faraday cages, so as to make the sensor simple and amenable to miniaturization.

## 2. Theory of Operation

The aerosol sensor is based on the fact that the average charge $\bar{q}$ per particle and the particle size *d_p_* have a certain exponential relationship [24] which can be expressed as follows:

(1) $$\bar{q}\left(d_{p}\right)=c\cdot {d_{p}}^{x}$$

where c is a constant determined through sensor calibration, *x* is a coefficient determined by the value of *N_i_*⋅*t_r_* (Fuchs theory). *N_i_* is the number concentration of ions in the charging section, *t_r_* is the exposure time of particles exposed to ions in the charging section. In general, the coefficient *x* is close to 1, which indicates a substantially linear relationship between the average charge and the particle size [1,25].

**Figure 1:**
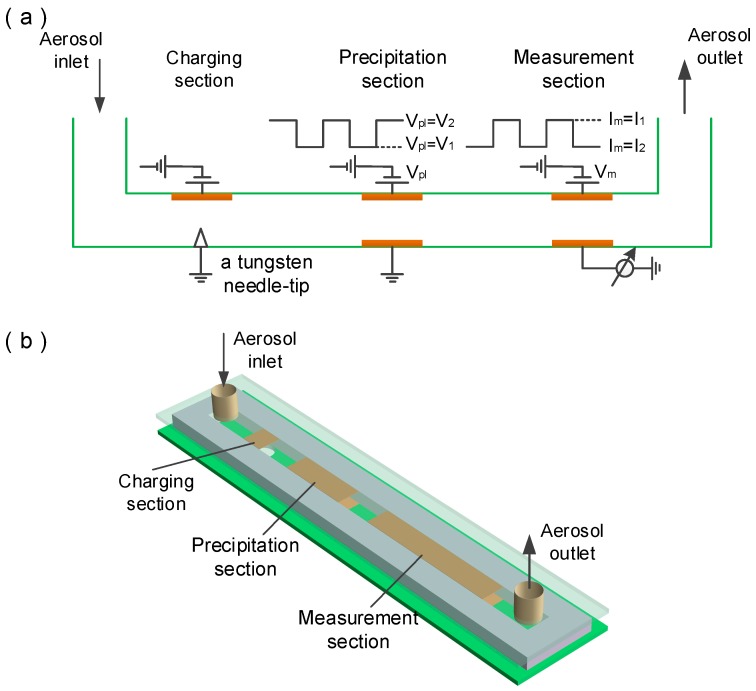
(**a**) The principle structure of the proposed aerosol sensor; and (**b**) Three-dimensional structure model diagram of the proposed aerosol sensor.

A schematic overview of the aerosol sensor is given in Figure 1. The aerosol sensor consists of three sections: charging section, precipitation section and measurement section, in each of which a pair of parallel electrodes are located and distributed along the flow channel. Firstly the aerosol particles and gas ion are electrically charged via diffusion charging in the charging section, where the charges are generated by a corona discharger with a tungsten needle-tip electrode loaded with a sufficiently high voltage (named corona discharge voltage) to ionize the surrounding air. Then the charged aerosol particles enter into the precipitation section, where a square signal with low and high voltages of *V*_1_ and *V*_2_ is imposed onto two opposite planar electrodes with a frequency of 0.1 Hz. At the *V*_1_ stage all gas ions are deposited on the planar electrodes in the precipitation section, and all charged aerosol particles pass through the precipitation section, deposit on the planar electrodes in the measurement section and export a total electrical current *I*_1_. At the *V*_2_ stage all gas ions and a part of the charged aerosol particles are deposited in the precipitation section, and the rest of the charged aerosol particles pass through the precipitation section, arrive at the measurement section and produce a total electrical current *I*_2_. The number concentration and the particle size can be figured out from the measurement currents *I*_1_ and *I*_2_ as follows [16]:

(2) $$N=S_{N}\cdot \left(I_{1}-I_{2}\right)$$

(3) $$d_{p}=S_{d}\cdot \frac{I_{1}}{I_{1}-I_{2}}$$

where *N* refers to the number concentration, *d_p_* refers to the particle size, *I*_1_ and *I*_2_ are the sensor output currents that correspond with the precipitating voltages *V*_1_ and *V*_2_, respectively. *S_N_* and *S_d_* are the constants dependent on the sensor geometry and working conditions.

## 3. Experimental Setup

**Figure 2:**
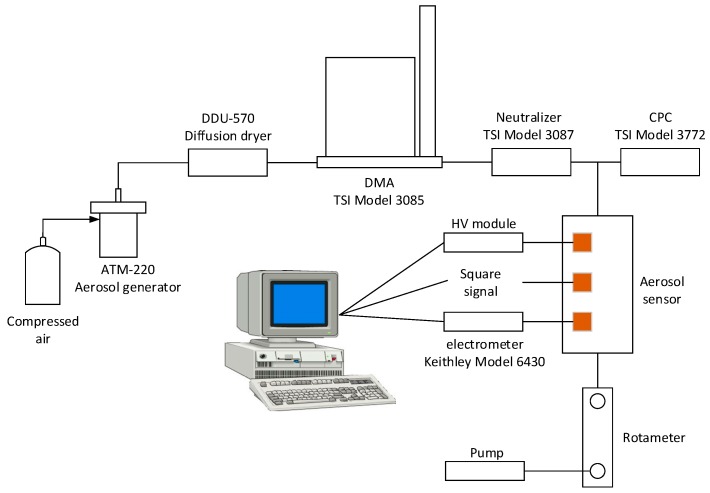
Schematic diagram of the experimental setup for testing the aerosol sensor.

Figure 2 shows the schematic diagram of the experimental setup for testing the developed aerosol sensor assessed with monodisperse airborne ultrafine particles. The experimental system involves an aerosol generator (ATM-220, TOPAS, Frankfurt, Germany), diffusion dryer (DDU-570, TOPAS), DMA (Model 3085, TSI, St. Paul, MN, USA), neutralizer (Model 3087, TSI), CPC (Model 3772, TSI), electrometer (Model 6430, Keithley, Cleveland, OH, USA) and the developed aerosol sensor. Polydisperse sodium chloride (NaCl) aerosol particles were generated by the aerosol generator, then the diffusion dryer absorbed excess water in the polydisperse aerosol. The DMA was used to select mono-sized aerosol particles according to their electrical mobility, and the charges existing in the aerosol particles were neutralized by the neutralizer to ensure all charged particles had been neutralized before entering into the aerosol sensor. The subsequent CPC accurately counted the number concentration. The selected monodisperse aerosol particles successively entered into the charging, precipitation and measurement sections of the aerosol sensor. The measurement currents were detected in real time by using the Keithley 6430 electrometer which has a peak-to-peak noise of 0.4 fA. The output frequency of the sensor was 0.1 Hz. The flow rate of the aerosol flow was set as 0.4 LPM. 

## 4. Preliminary Experiments 

In order to determine the number concentration and particle size of monodisperse aerosol particles, a series of preliminary experiments needed to be conducted. The first preliminary experiments were conducted to determine the optimal working conditions for the aerosol sensor. The corona discharge voltage, the square voltages applied on the precipitation electrodes, and the voltage imposed on the measurement electrodes for collecting the charge current were tested and optimized. Firstly the corona discharge voltage of the aerosol sensor was determined by gradually increasing the voltage on the charging section until the corona discharge occurred and self-sustained. Then, the discharge voltage was maintained. The measurement voltage imposed on the measurement electrodes was determined by increasing the magnitude of the measurement voltage until the charge current achieved saturation while the precipitation electrodes were floating, which ensured all charged aerosol particles to be detected could be collected by the measurement section. The square voltages applied on the precipitation electrodes was determined by the criteria that the low voltage was used for depositing the excess gas ions except the charged aerosol particles onto the precipitation section and the high voltage was for depositing a part of the charged aerosol particles onto the precipitation section. For our developed sensor, the corona voltage was finally set as 1400 V, the low and high voltages of the precipitation square signal were set as 0.9 V and 3 V, respectively, while the voltage imposed on the measurement electrodes was −5 V.

The second experiments were conducted to determine the initial parameters for the aerosol sensor. Two conditions with and without diffusion charging were tested, respectively. In the experiments, pure air without an aerosol was used as the gas medium and the precipitation section was switched off. The experimental results are shown in Table 1, where the currents were collected at the measurement section when the charging section was turned off and on, respectively. The initial parameters were used to test the initial condition of the sensor, for example the average current under air without charging indicates the leakage current of the sensor.

## 5. Results and Discussion

**Figure 3:**
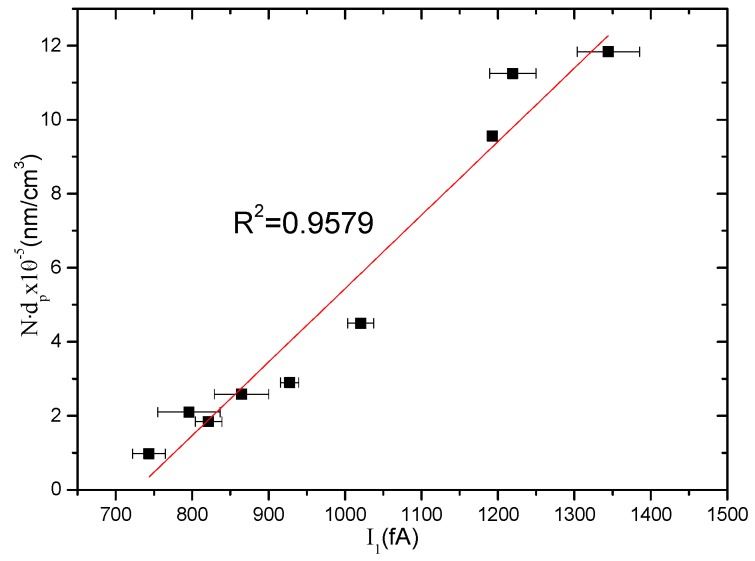
The relationship between *N*⋅*d_p_* and *I*_1_ for the measurement of monodisperse aerosols with particle sizes in the range of 50–253 nm and number concentrations in the range of 300–2.5 × 10^4^ /cm^3^.

Monodisperse NaCl aerosol particles with particle sizes in the range of 50–253 nm and number concentrations in the range of 300–2.5 × 10^4^ /cm^3^ were measured by using the developed aerosol sensor. Figure 3 shows the results of the tested current *I*_1_ as a function of the particles’ value of *N*⋅*d_p_*. Figure 3 indicates a linear relationship between *I*_1_ and *N*⋅*d_p_* with a correlation coefficient of *R*^2^ = 0.9579. The results are in good agreement with a previous report [17]. 

**Figure 4:**
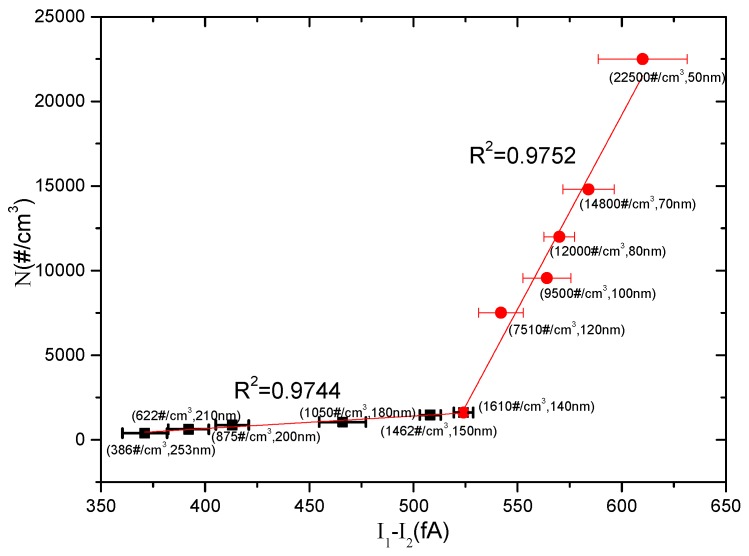
The relationship between *N* and *I*_1__ − _*I*_2_ for testing monodisperse aerosols with diameters in the range of 50–253 nm.

**Figure 5:**
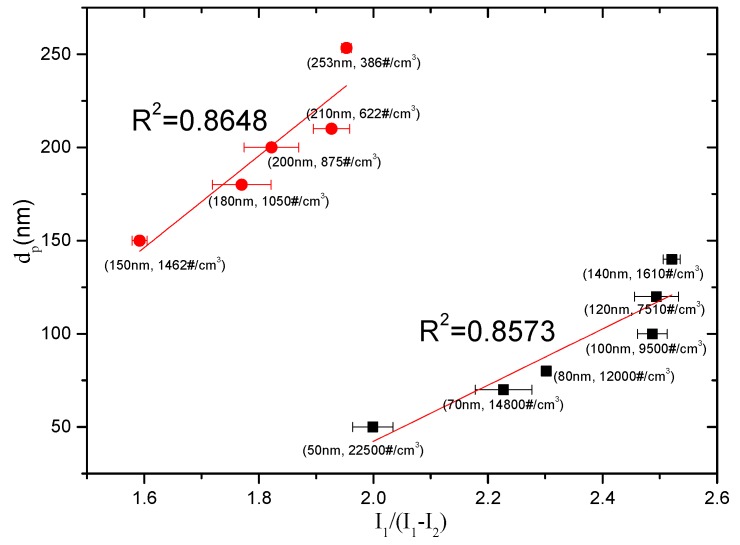
The relationship between *d_p_* and *I*_1_/(*I*_1_ − *I*_2_) for testing monodisperse aerosols with number concentrations in the range of 300–2.5 × 10^4^ /cm^3^.

Figure 4 and Figure 5 show the relationship between the particle number concentration *N* and the measured *I*_1_ − *I*_2_, and the relationship between the aerosol particle size *d_p_* and the measured *I*_1_/(*I*_1_ − *I*_2_), respectively. The results indicate that the size domain displayed a turning point at *d_p_* = 150 nm. The number concentration *N* exhibited different linear relationships with the measured *I*_1_ − *I*_2_ in two regions of 50 < *d_p_* < 150 nm and 150 < *d_p_* < 253 nm, respectively. The relationship between the particle size *d_p_* and the measured *I*_1_/(*I*_1_ − *I*_2_) was similar. Linear fits were conducted for the results and the relationships could be expressed as follows:

For 50 < *d_p_* < 150 nm:

(4) $$N=229.49\cdot \left(I_{1}-I_{2}\right)-118484$$

(5) $$d_{p}=150.81\cdot \frac{I_{1}}{I_{1}-I_{2}}-259.31$$

For 150 < *d_p_* < 253 nm:

(6) $$N=7.4064\cdot \left(I_{1}-I_{2}\right)-2300$$

(7) $$d_{p}=246.2\cdot \frac{I_{1}}{I_{1}-I_{2}}-247.63$$

The change at the turning point of *d_p_* = 150 nm might be attributed to the variation of the particle flow behavior. The Knudsen number $K_{n}=2\bar{\lambda }/d_{p}$, referring to the ratio of the gas’ molecular mean free path to the particle dimension, determines the type of particle flow. At normal temperature and pressure (NTP), the gas molecular mean free path is $\bar{\lambda }=66.4\;\text{nm}$ [1]. It is presumed that at *d_p_* < 150 nm the aerosol particle flow presents a slip flow regime, but a continuum regime flow at *d_p_* > 150 nm. The transformation of the flow behavior affects the particle diffusion charging and thus changes the relationships between the measurement currents and the particle parameters [19].

**Figure 6:**
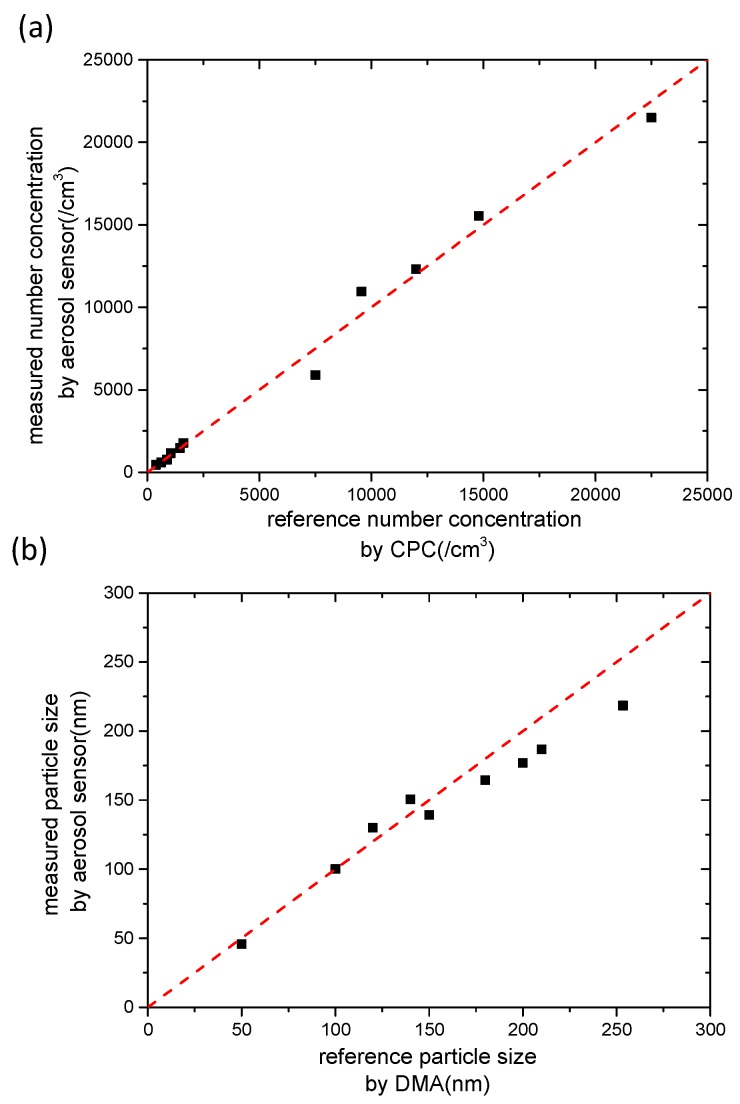
(**a**) Comparison of results measured by the aerosol sensor and reference data determined by CPC for number concentration; and (**b**) comparison of results measured by the aerosol sensor and reference data determined by DMA for particle size.

Based on the abovementioned analysis, the aerosol sensor figures out the measured aerosol number concentration from the relationship between *N* and *I*_1_ − *I*_2_ shown in Figure 4, and then derives the measured particle size from the relationship between *N*⋅*d_p_* and *I*_1_ shown in Figure 3. The comparison between the measured results by the aerosol sensor and the reference data are shown in Figure 6, where the reference data of the number concentration and the particle size were detected by CPC and DMA, respectively. 

Table 2 lists the mean square deviations of the sensor results from the reference data for the number concentration and particle size, which were 6.7% and 3.8%, respectively. The experimental results proved the effectiveness of the aerosol sensor for detecting the number concentration and the particle size of monodisperse ultrafine aerosols. Actually the developed aerosol sensor can detect not only monodisperse aerosol particles but also polydisperse aerosol particles with normal distribution based on the same diffusion charging theory. The detected particle size is the number-averaged particle diameter of the polydisperse aerosol. For measuring polydisperse aerosol particles, a calibration experiment using standard polydisperse particles with a normal distribution is required prior to the particle detection. Compared with existing instruments [26,27] using different particle types (e.g., NaCl, PSL, DOP) and other on-field micro-aerosol sensors, some features of the performance of the sensor still needs to be improved on, such as measurement accuracy, miniaturization, and applications with polydisperse particles.

## 6. Conclusions

This paper has presented a micro-aerosol sensor based on diffusion charging and electrical detection to measure number concentrations of 300–2.5 × 10^4^ /cm^3^ and particle sizes of 50–253 nm. The aerosol sensor is composed of planar electrodes printed on two circuit boards assembled in parallel, which integrate charging, precipitating and measurement elements into one chip. The device is simple and practical for handheld instruments. The comparison between the results of the proposed aerosol sensor and the reference data of the measured number concentration and particle size of monodisperse aerosol presented a satisfactory result and proved the effectiveness of the aerosol sensor for detecting ultrafine aerosol particles. In the future, we will further improve the performances of the sensor on such as measurement accuracy, miniaturization, and applications to polydisperse particles.

## Figures and Tables

**Table 1:** The initial parameters for the aerosol sensor.

Conditions	Average Current (fA)	Standard Deviation (fA)
Air without charging	−41.4	−1.4
Air with diffusion charging	−83.7	−7.6

**Table 2:** The deviations of the measured results by aerosol sensor from reference data.

Parameter	Mean Square Deviation	Maximum Deviation	Minimum Deviation
Number concentration	6.7%	21%	0.03%
Aerosol particle size	3.8%	13.8%	0.1%
